# Supplementary material for: Design of effective value calculation model for dynamic dataflow of infrared gas online monitoring
Source: PLoS One. 2021 Oct 28;16(10):e0259155. doi: 10.1371/journal.pone.0259155 (PMC8553131; doi:10.1371/journal.pone.0259155)
Supplement: S1 Definition — (DOCX) [file pone.0259155.s005.docx]

**Definition**

**Dynamic data flow:** when a gas sensor is working, it analyzes the target gas concentration in its gas chamber in real time, and continuously send the value to microcomputer, forming a data flow.

**Effective value / effective data：**the value can indicate the tested parameter in a series of process data when a sensor has a long response time.

**Invalid value/invalid data:** process data that approximates the effective value from the initial value.

**GC value:** CH_4_ and CO_2_ concentration analyzed by gas chromatography.

**Data Numerical Distribution (DND):** the absolute value of the difference between adjacent data in one monitoring window data set.

**Data Frequency Distribution of Subdataset (DFDS):** the amount of data in every subdataset when divided the sensor value from C_t_ to the maximum into several equal parts in one monitoring window data set.

**Effective Segmentation Statistical Set:** Based on DAW dataset segmentation, statistical calculation of the average value of each subdataset and DFDS value. This data set is a dimensionality reduction calculation of the DAW data set.
