# Supplementary material for: Design of effective value calculation model for dynamic dataflow of infrared gas online monitoring
Source: PLoS One. 2021 Oct 28;16(10):e0259155. doi: 10.1371/journal.pone.0259155 (PMC8553131; doi:10.1371/journal.pone.0259155)
Supplement: Attachment — Submitted filename: PONE-D-20-38017 review comments.docx [file pone.0259155.s006.docx]

**Design of Effective Value Calculation Model for Dynamic Dataflow of Infrared Gas Online Monitoring**

This study attempts to explain design of effective value calculation model for dynamic dataflow of infrared gas online monitoring. The study develops an effective value calculation model using the method of dimensionality reduction of dynamic data. The model was based on the distribution characteristics of the process data, and consisted of four steps. This research aids in improving knowledge on gas monitoring and should be considered for publication after addressing the following suggestions / comments:

1. The introduction section of the study should include the background of the study and the concept of dynamic data flow analysis for clarity on the subject. This is necessary to offer an understanding of the topic and scope of the study.
2. The results and discussion section should be enhanced further. In fact, it primarily offers a summary and repetition of information already appearing in both introduction and methods section. Therefore, this section needs to be revised to offer a discussion of the study results.
3. The study lacks conclusion section. This is an integral part of any research and need to be added.
4. Additionally, the following style and grammatical errors should be addressed:
i. Check the in-text references and make sure they are according to the author guideline. Avoid any mixing two different referencing styles. For example, in line 71 there is a superscript 9. Is this for the foot notes or a typo?
ii. Ensure that you use correct symbols all through the paper. Example, check line 103
iii. Follow the provided author guidelines while numbering and formatting your equations
iv. Kindly proof read your document before submission to ensure that all sentences are well structured.
